# *Brachymna tenuis* Stål, 1861 (Hemiptera: Pentatomidae), a new invasive bamboo pest in Korea with notes on insects associated with bamboos

**DOI:** 10.3897/BDJ.8.e58476

**Published:** 2020-11-30

**Authors:** Soojeong Ahn, WonGun Kim, Sangsu Kim, Geonho Cho

**Affiliations:** 1 Masan University 9-310, Changwon-si, Gyeongsangnam-do, South Korea Masan University 9-310 Changwon-si, Gyeongsangnam-do South Korea; 2 Dogok Rexle Apt. 207-404, Seolleung-ro 221, Gangnam-gu, Seoul, South Korea Dogok Rexle Apt. 207-404, Seolleung-ro 221, Gangnam-gu Seoul South Korea; 3 Gichan Engineering Co. Ltd., Naju-si, 55258, Jeollanam-do, South Korea Gichan Engineering Co. Ltd. Naju-si, 55258, Jeollanam-do South Korea; 4 Research Institute of Basic Sciences, Seoul National University, 08826, Seoul, South Korea Research Institute of Basic Sciences, Seoul National University, 08826 Seoul South Korea

**Keywords:** bamboo, invasive, pest, distribution, *
Brachymna
*, Korea

## Abstract

**Background:**

We report first observations of the invasive bamboo pest, *Brachymna tenuis* Stål, 1861 in Korea as the first species of *Brachymna* Stål, 1861 (Pentatomidae) reported from the country.

**New information:**

Comments on its pest status and distribution are provided. General information on this bamboo-feeding insect in Korea is analysed and provided for the first time.

## Introduction

Bamboo grows in the tropics and subtropics and has received increasing attention because of its fast-growing nature, social usage and industrial demand, for example, foods, crafts, building material and energy resource (Kim et al. 2018). In Korea, there are 18 species of bamboos distributed mostly in the southern part of the country (Kong 1985). Amongst them, eleven dominant bamboo species either occur naturally or are planted and they occupy more than 22 thousand hectares (Kim et al. 2018). The number of insects that feed on bamboos is estimated to be more than 1200 insects in the world (Shu and Wang 2015). The numbers may vary depending on the country and province, ranging from 97 to 244 insect species feeding on bamboos in China (Huang and Liu 2006, Zhao et al. 2006, Zhang and Zuo 2015). In Korea, the review of the entire bamboo-feeding insects has never been done before.

Bamboo pest, *Brachymna tenuis* Stål, 1861 and its synonym, *Balsa extenuata* Walker, 1867 were described from Hong Kong and the Chinese mainland, respectively and subsequent records were made from twelve Provinces in China (Hoffmann 1932, Hsiao and Zheng 1977, Rider et al. 2002, Aukema and Rieger 2006, Huang and Liu 2006, Zhao et al. 2006, Zhang et al. 2008, Zhang and Zuo 2015), Japan (Aukema and Rieger 2006, Ishikawa et al. 2012, Tanaka 2013, Tanaka 2014, Igasaki 2016, Igasaki 2017, Igasaki 2018, Kanetada 2017) and Taiwan (Taiwan Encyclopedia of Life 2018). Recently, the first three authors observed somewhat large, exotic, invasive, bamboo-feeding stink bugs from southern Korea, which had never been recorded. The aim of the present paper is to report new records of invasive bamboo pest *Brachymna tenuis* from Korea, to discuss the distribution of the species and to provide, for the first time, a list of bamboo-feeding insects in Korea, based on a literature review.

## Materials and methods

Surveys were conducted in natural and cultivated bamboos, located in the southern Provinces of South Korea (Gyeongsangnam-do and Jeollanam-do) in 2020. Adults and nymphs were observed by visual inspection. The specimens were morphologically identified using the reference books by Lin and Zhang (1993) and Ishikawa et al. (2012). Photographs of habitus were taken using a DSLR camera (Nikon D500, D7100, Nikon 60 mm Micro). The plant names follow The Plant List (2016). The examined specimens were deposited in the College of Agriculture and Life Science, Seoul National University (SNU), Seoul and the private collections of the authors.

## Taxon treatments

### Brachymna tenuis

Stål, 1861

D8F7725B-3CC7-521B-A965-5E3BBA631399

http://taieol.tw/data_objects/93100

PRJNA550733

#### Materials

- **Type status:** Other material. **Occurrence:** recordedBy: S. Ahn; W.G. Kim; individualCount: 16; sex: 3 males, 3 females, 10 nymphs; lifeStage: 6 adults, 10 nymphs; establishmentMeans: invasive; occurrenceStatus: present; preparations: in 95% ethanol; disposition: in collection; **Taxon:** scientificName: Brachymna tenuis; order: Hemiptera; family: Pentatomidae; genus: Brachymna; specificEpithet: tenuis; taxonRank: species; nomenclaturalCode: ICZN; **Location:** higherGeography: East Asia; South Korea; country: South Korea; stateProvince: Gyeongsangnam-do; municipality: Jinju-si; locality: Gajwa-dong, San 113-1; **Identification:** identifiedBy: S. Ahn; W.G. Kim; S. Kim; G. Cho; dateIdentified: 2020-07; **Event:** samplingProtocol: visual inspection; eventDate: 2020-06-11; year: 2020; month: 6; day: 11; habitat: *Semiarundinaria* sp.; **Record Level:** language: en; institutionCode: SNU; basisOfRecord: PreservedSpecimen
- **Type status:** Other material. **Occurrence:** recordedBy: S. Ahn; individualCount: 2; sex: 2 nymphs; lifeStage: 2 nymphs; establishmentMeans: invasive; occurrenceStatus: present; preparations: in 95% ethanol; disposition: in collection; **Taxon:** scientificName: Brachymna tenuis; order: Hemiptera; family: Pentatomidae; genus: Brachymna; specificEpithet: tenuis; taxonRank: species; nomenclaturalCode: ICZN; **Location:** higherGeography: East Asia; South Korea; country: South Korea; stateProvince: Gyeongsangnam-do; municipality: Haman-gun; locality: Beopsu-myeon, Jumul-ri, San 1; **Identification:** identifiedBy: S. Ahn; W.G. Kim; S. Kim; G. Cho; dateIdentified: 2020-07; **Event:** samplingProtocol: visual inspection; eventDate: 2020-06-25; year: 2020; month: 6; day: 25; habitat: *Semiarundinaria* sp.; **Record Level:** language: en; institutionCode: SNU; basisOfRecord: PreservedSpecimen
- **Type status:** Other material. **Occurrence:** recordedBy: S. Ahn; individualCount: 3; sex: 3 nymphs; lifeStage: 3 nymphs; establishmentMeans: invasive; occurrenceStatus: present; preparations: in 95% ethanol; disposition: in collection; **Taxon:** scientificName: Brachymna tenuis; order: Hemiptera; family: Pentatomidae; genus: Brachymna; specificEpithet: tenuis; taxonRank: species; nomenclaturalCode: ICZN; **Location:** higherGeography: East Asia; South Korea; country: South Korea; stateProvince: Gyeongsangnam-do; municipality: Changwon-si; locality: Masanhoewon-gu, Guam-dong 669-9; **Identification:** identifiedBy: S. Ahn; W.G. Kim; S. Kim; G. Cho; dateIdentified: 2020-07; **Event:** samplingProtocol: visual inspection; eventDate: 2020-06-28; year: 2020; month: 6; day: 28; habitat: *Semiarundinaria* sp.; **Record Level:** language: en; institutionCode: SNU; basisOfRecord: PreservedSpecimen
- **Type status:** Other material. **Occurrence:** recordedBy: S. Ahn; individualCount: 4; sex: 1 male, 3 nymphs; lifeStage: 1 adult, 3 nymphs; establishmentMeans: invasive; occurrenceStatus: present; preparations: in 95% ethanol; disposition: in collection; **Taxon:** scientificName: Brachymna tenuis; order: Hemiptera; family: Pentatomidae; genus: Brachymna; specificEpithet: tenuis; taxonRank: species; nomenclaturalCode: ICZN; **Location:** higherGeography: East Asia; South Korea; country: South Korea; stateProvince: Gyeongsangnam-do; municipality: Jinju-si; locality: Gajwa-dong 952-1; **Identification:** identifiedBy: S. Ahn; W.G. Kim; S. Kim; G. Cho; dateIdentified: 2020-07; **Event:** samplingProtocol: visual inspection; eventDate: 2020-07-11; year: 2020; month: 7; day: 11; habitat: *Semiarundinaria* sp.; **Record Level:** language: en; institutionCode: SNU; basisOfRecord: PreservedSpecimen
- **Type status:** Other material. **Occurrence:** recordedBy: S. Ahn; individualCount: 1; sex: 1 nymph; lifeStage: 1 nymph; establishmentMeans: invasive; occurrenceStatus: present; preparations: in 95% ethanol; disposition: in collection; **Taxon:** scientificName: Brachymna tenuis; order: Hemiptera; family: Pentatomidae; genus: Brachymna; specificEpithet: tenuis; taxonRank: species; nomenclaturalCode: ICZN; **Location:** higherGeography: East Asia; South Korea; country: South Korea; stateProvince: Gyeongsangnam-do; municipality: Hadong-gun; locality: Yangbo-myeon, Jangam-ri 419; **Identification:** identifiedBy: S. Ahn; W.G. Kim; S. Kim; G. Cho; dateIdentified: 2020-07; **Event:** samplingProtocol: visual inspection; eventDate: 2020-07-15; year: 2020; month: 7; day: 15; habitat: *Miscanthus sinensis* Andersson; **Record Level:** language: en; institutionCode: SNU; basisOfRecord: PreservedSpecimen

#### Diagnosis

According to Lin and Zhang (1993), *Brachymna tenuis* resembles *B. castanea*. It differs from the latter by the yellowish-brown body colour (Fig. 1a, b) (ferruginous in *B. castanea*) and the pygophore bearing dentate postero-lateral processes that are obtusely curved from the postero-ventral margin (Fig. 1c) (postero-lateral processes make approximately a right angle with the postero-ventral margin in *B. castanea*).

#### Distribution

China, Japan, Taiwan and South Korea (new record).

## Analysis

Provisionally, 61 species are associated with bamboos in South Korea with the majority of the insects belonging to sap-sucking Hemiptera (52.46%). They are mainly polyphagous and include mostly aphids and scale insects. Lepidoptera are the second largest group, making up 31.15% of the total; these include many defoliators, leaf-rollers and one bamboo-shoot cutworm (*Bambusiphila vulgaris*). Coleoptera are the third order, with 14.75% of the species, which include deleterious bamboo borers, such as *Chlorophorus annularis* (Cerambycidae) and *Dinoderus* spp. (Bostrichidae). The bamboo-feeding sawfly *Tenthredo nigropicta* (Tenthredinidae) is included in the list as the only member of Hymenoptera (Table 1).

**Figure 3.**
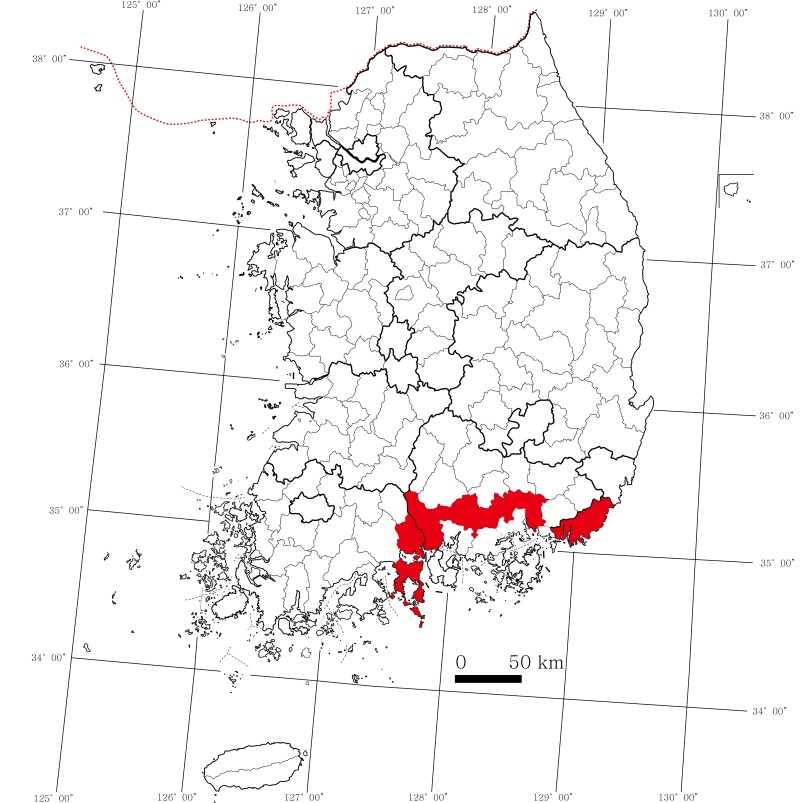
Map showing the distribution of *Brachymna tenuis* in South Korea.

The invasive stink bug was identified as *Brachymna tenuis* Stål, 1861 (Hemiptera: Heteroptera: Pentatomidae) that is recorded in South Korea for the first time (Figs 1, 2). It is one of the most important pest species feeding on bamboo branch and culm. A heavy infestation may cause defoliation, wilting of young shoots and branches and even death of the culm (Shu and Wang 2015). The species is polyphagous and is reported from various bamboo species in South Korea, for example, *Phyllostachys elegans* McClure and *Semiarundinaria densiflora* (Rendle) T.H.Wen (Poaceae). Sometimes, it feeds also on *Miscanthus sinensis* Andersson (Poaceae). Numerous adults and nymphs were observed on planted bamboos in the urban areas of Korean southern provinces. The species was also observed in Busan-si, Gwangyang-si and Yeosu-si without detailed collection data (Fig. 3).

## Discussion

In East Asia, *Brachymna tenuis* is widespread in many tropical and subtropical Chinese Provinces (Anhui, Fujian, Guangdong, Guizhou, Henan, Hong Kong, Hubei, Hunan, Jiangsu, Jiangxi, Sichuan, Yunnan and Zhejiang) (Hoffmann 1932, Hsiao and Zheng 1977, Rider et al. 2002, Aukema and Rieger 2006, Huang and Liu 2006, Zhao et al. 2006, Zhang et al. 2008, Zhang and Zuo 2015) and it has been reported from subtropical Japanese Prefectures (Ryukyu, Oita and Yamaguchi) (Aukema and Rieger 2006, Tanaka 2013, Tanaka 2014, Igasaki 2016, Igasaki 2017, Igasaki 2018, Kanetada 2017) and Taiwan (Taiwan Encyclopedia of Life 2018). In Japan, after the first observation of the species in 1997 (Ishikawa et al. 2012), recent range expansion to the north and abnormal outbreaks have been observed (Tanaka 2013, Tanaka 2014, Igasaki 2017, Igasaki 2018). In South Korea, it was first found in 2020 on planted bamboos in the southern provinces (present paper). Since 2010, the first three authors carried out extensive field monitoring research mainly on terrestrial Hemiptera, but they had not seen any *Brachymna* species before. As the adults of the species have been found along with numerous nymphs in restricted areas, we concluded that the species was very recently introduced and established into the country. Little is known about the viability and host plant usage of the species in South Korea. More attention is required in order to limit the further spread of the pest species.

## Supplementary Material

XML Treatment for Brachymna tenuis

## Figures and Tables

**Figure 1a.**
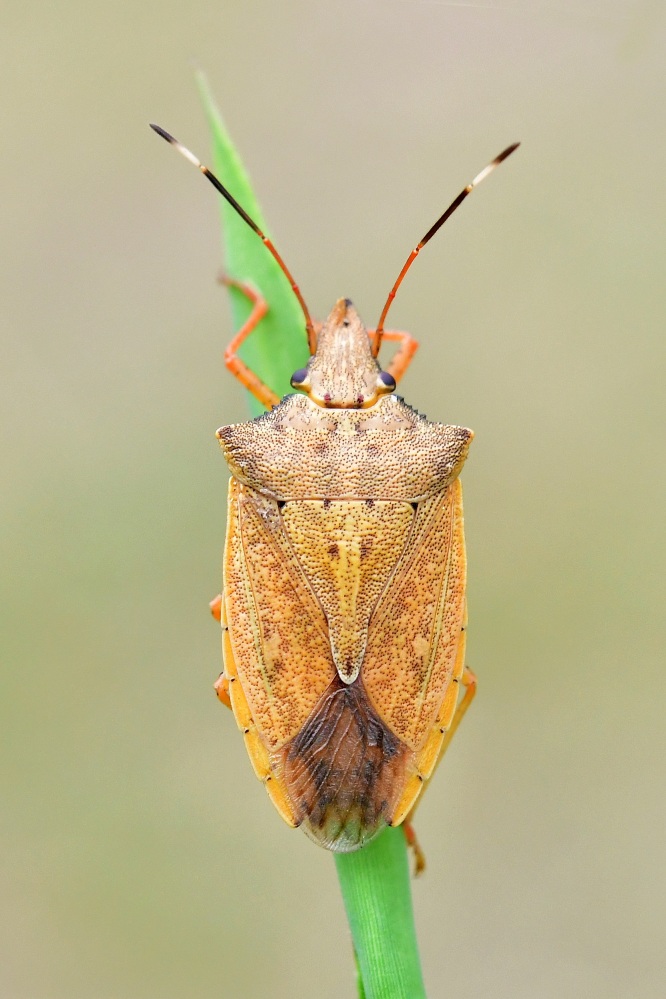
Adult male, dorsal view.

**Figure 1b.**
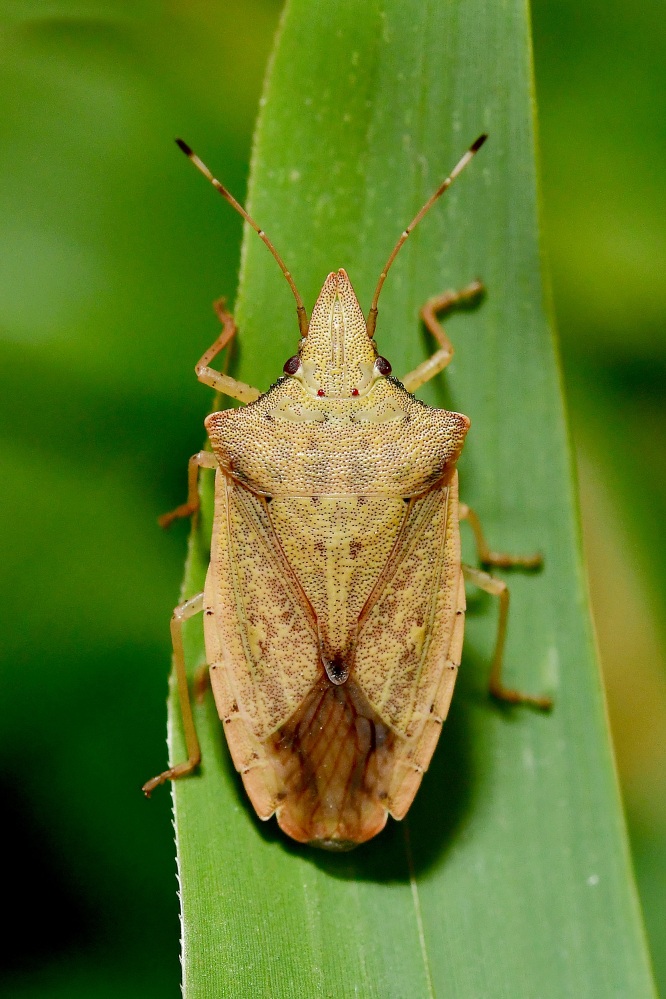
Adult female, dorsal view.

**Figure 1c.**
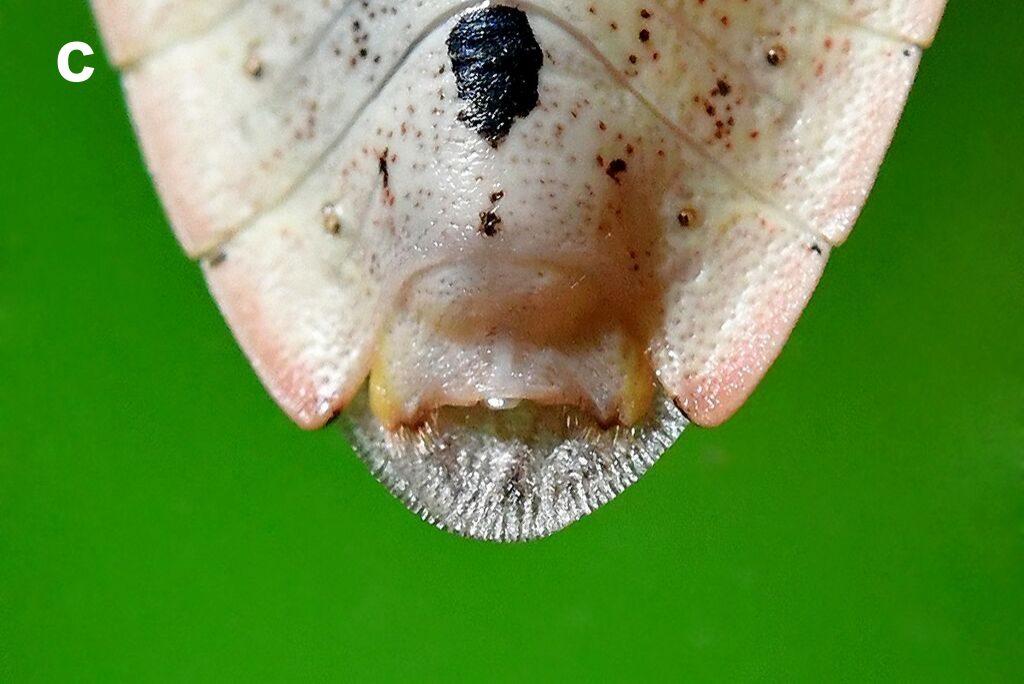
Apex of abdomen of male, ventral view.

**Figure 1d.**
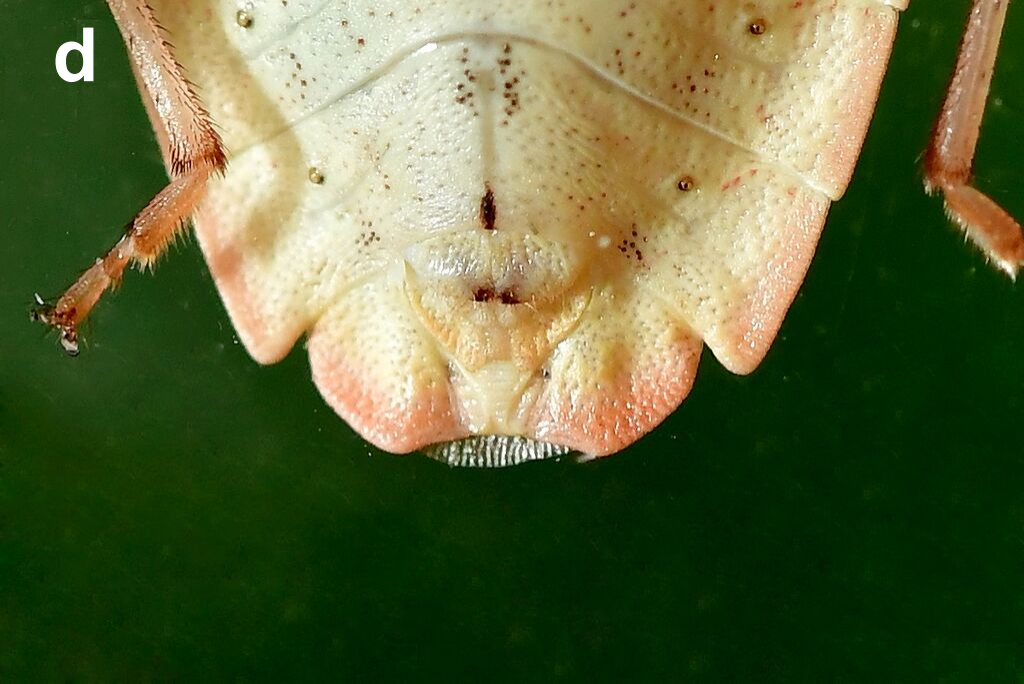
Apex of abdomen of female, ventral view.

**Figure 2a.**
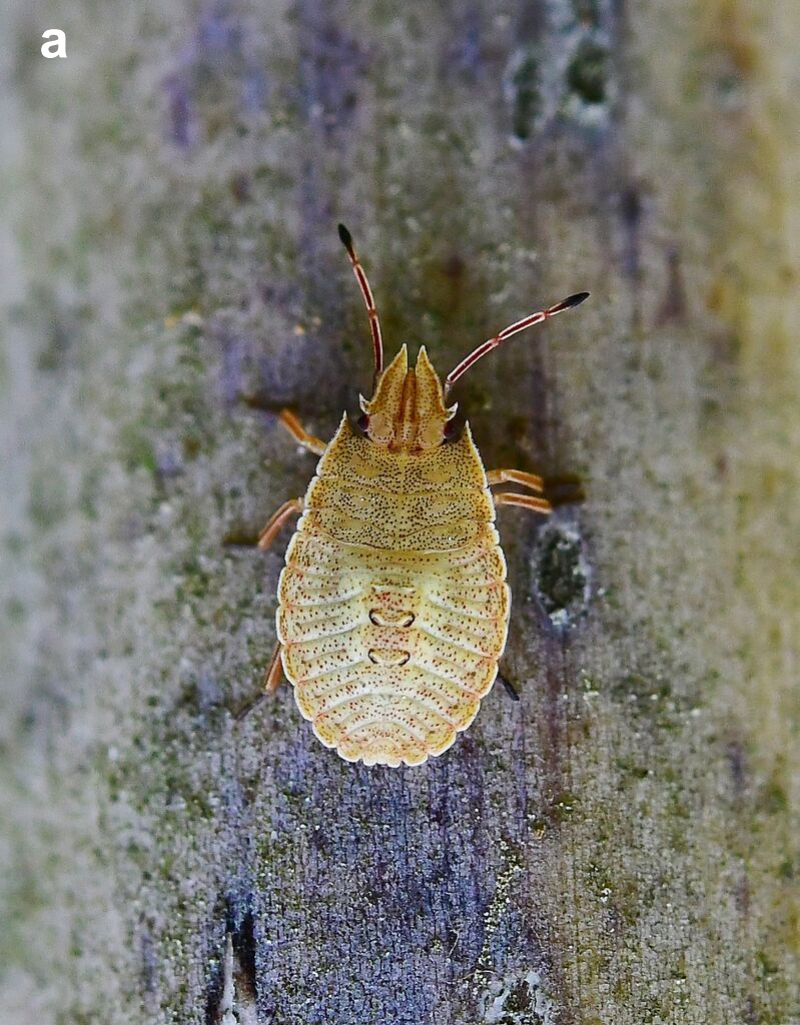
Third instar nymph.

**Figure 2b.**
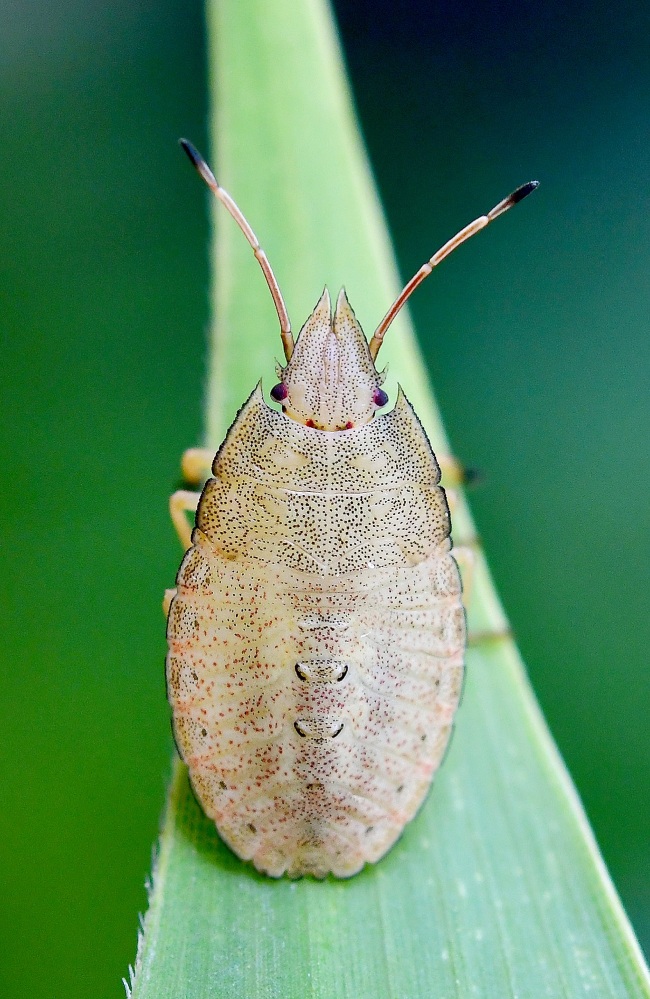
Fourth instar nymph.

**Figure 2c.**
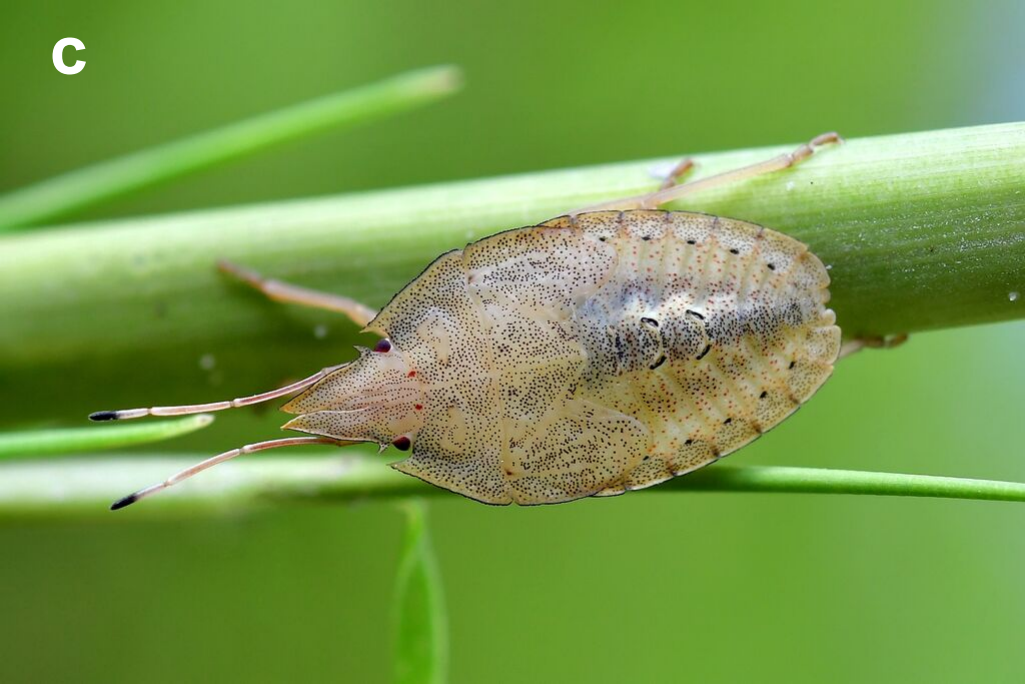
Fifth instar nymph.

**Table 1.** Provisional list of insect species feeding on bamboos in Korea. The species that possibly damages bamboo is marked with *.

Insect	Host plants with reference	Feeding habits with reference
** COLEOPTERA **		
** Bostrichidae **		
*Dinoderus japonicus* Lesne, 1895	*Phyllostachys*, *Pleioblastus* (Bieńkowski and Orlova-Bienkowskaja 2017)	Borer (Bieńkowski and Orlova-Bienkowskaja 2017)
*Dinoderus minutus* (Fabricius, 1775)	*Bambusa* spp., *Dendrocalamus* spp., *Phyllostachys* spp. (Watanabe et al. 2018, CABI 2020)	Borer (Mori and Arai 1979, Watanabe et al. 2018, CABI 2020)
*Lyctus brunneus* (Stephens, 1830)	Bamboo (Liu and Geis 2019)	Borer (Mori and Arai 1979)
** Cerambycidae **		
*Bumetopia oscitans* Pascoe, 1858	*Arundinaria simonii* (Park 2015)	Borer (Park 2015)
*Chlorophorus annularis* (Fabricius, 1787)	Bamboos; *Bambusa* spp., *Chimonobambusa tumidissinoda*, *Dendrocalamus strictus*, *Indosasa crassiflora*, *Phyllostachys* spp., *Sinobambusa gibbosa*, *Sinocalamus* spp. (Mori and Arai 1979, Hill 2008, Lim et al. 2014, Suma and Bella 2018)	Borer (Mori and Arai 1979, Hill 2008, Suma and Bella 2018)
*Purpuricenus temminckii* Guerin-Meneville, 1844	*Phyllostachys* spp., *Sasa* spp. (Mori and Arai 1979, Lim et al. 2014)	Borer (Mori and Arai 1979)
*Niphona furcata* (Bates, 1873)	*Phyllostachys*, *Pleioblatus*, *Pseudosasa japonica*, *Sasa* spp. (Haoje et al. 2002, Lim et al. 2014)	Borer (Haoje et al. 2002)
** Nitidulidae **		
*Epuraea submicrurula* Reitter, 1875	*Sasa* spp. (Sakata et al. 2020) (North Korea)	Florivory (Sakata et al. 2020)
** Ptinidae **		
*Oligomerus japonicus* Sakai, 1982*	Unknown	Unknown
** HEMIPTERA **		
** Aclerdidae **		
*Nipponaclerda biwakoensis* (Kuwana, 1907)	*Sasa borealis* (García-Morales et al. 2016, Suh 2020)	Sap-sucking
** Alydidae **		
*Distachys unicolor* (Scott, 1874)	*Sasa borealis* (Ahn et al. 2018)	Sap-sucking
*Distachys vulgaris* Hsiao, 1964	*Sasa borealis* (Ahn et al. 2018)	Sap-sucking
** Asterolecaniidae **		
*Bambusaspis bambusicola* (Kuwana, 1916)	*Bambusa* spp., *Phyllostachys* spp. (García-Morales et al. 2016, Suh 2020)	Sap-sucking
** Aphididae **		
*Ceratoglyphina styracicola* (Takahashi, 1921)	Bamboos (*Arundinaria* or *Pleioblastus*) (Blackman and Eastop 2020)	Sap-sucking
*Ceratovacuna cerbera* Aoki, Kurosu, Shin & Choe, 1999	*Sasa* spp. including *S. borealis* and *S. veichii* (Aoki et al. 1999, Aoki and Kurosu 2010, Blackman and Eastop 2020)	Sap-sucking
*Ceratovacuna japonica* (Takahashi, 1924)	Small bamboos (*Arundinaria*, *Bambusa*, *Sasa* sp.) (Blackman and Eastop 2020)	Sap-sucking
*Melanaphis bambusae* (Fullaway, 1901)	Bamboos (*Arundinaria*, *Bambusa*, *Phyllostachys*) (Blackman and Eastop 2020)	Sap-sucking
*Paracolopha morrisoni* (Baker, 1919)	Bamboos (*Arundinaria*, *Phyllostachys*, *Sasa* spp.) (Blackman and Eastop 2020)	Sap-sucking
*Rhopalosiphum rufiabdominale* (Sasaki, 1899)	Numerous species of Poaceae (Blackman and Eastop 2020)	Sap-sucking
*Takecallis alba* Lee, 2018	*Pseudosasa* sp., *Sasa* spp. (Lee and Lee 2018, Blackman and Eastop 2020, Rakhshani et al. 2020)	Sap-sucking
*Takecallis arundicolens* (Clarke, 1903)	*Arundinaria* spp., *Bambusa* spp., *Phyllostachys* spp., *Pleioblastus chino*, *Pseudosasa japonica*, *Sasa* spp., *Sasaella ramosa* (Lee and Lee 2018, Blackman and Eastop 2020, Rakhshani et al. 2020)	Sap-sucking
*Takecallis arundinariae* (Essig, 1917)	*Arundinaria* spp., *Bambusa* spp., *Dendrocalamus* spp., *Phyllostachys* spp., *Pseudasasa japonica*, *Sasa* spp., *Sinoarundinaria reticulata*, *Sinobambusa tootsik*) (Lee and Lee 2018, Blackman and Eastop 2020, Rakhshani et al. 2020)	Sap-sucking
*Takecallis taiwana* (Takahashi, 1926)	*Arundinaria* spp., *Bambusa* spp., *Dendrocalamus asper*, *Phyllostachys* spp., *Pleioblastus* spp., *Sasa* spp., *Shibataea kumasana*) (Lee and Lee 2018, Blackman and Eastop 2020, Rakhshani et al. 2020)	Sap-sucking
** Blissidae **		
*Dimorphopterus japonicus* (Hidaka, 1959)	*Chimonobambusa marmorea*, *Sasa senanensis* (Sakata et al. 2020)	Florivory, sap-sucking (Sakata et al. 2020)
** Cicadellidae **		
*Scaphoideus festivus* Matsumura, 1902	Bamboo (Yang et al. 2013)	Sap-sucking
** Coccidae **		
*Coccus hesperidum* Linnaeus, 1759	Various plants of 346 genera in 121 families including *Bambusa vulgaris* (García-Morales et al. 2016, Choi and Lee 2018)	Sap-sucking
** Diaspididae **		
*Kuwanaspis hikosani* (Kuwana, 1902)	*Arundinaria simonii*, *Bambusa* spp., *Phyllostachys* spp., *Sasa* sp.) (Suh and Hodges 2007, García-Morales et al. 2016, Malumphy and Salisbury 2016, Suh 2020)	Sap-sucking
*Kuwanaspis howardi* (Cooley, 1898)	*Arundinaria*, *Bambusa* spp., *Fargesia nitida*, *Phyllostachys* spp. (Suh and Hodges 2007, García-Morales et al. 2016, Malumphy and Salisbury 2016, Suh 2020)	Sap-sucking
*Kuwanaspis pseudoleucaspis* (Kuwana, 1902)	*Arundinaria* spp., *Bambusa*, *Drepanostachyum*, *Fargesia*, *Himalayacalamus*, *Phyllostachys* spp., *Pleioblastus*, *Pseudosasa japonica*, *Sasa* spp., *Semiarundinaria*, *Sinobambusa* spp. (Suh and Hodges 2007, García-Morales et al. 2016, Suh 2020)	Sap-sucking
*Odonaspis secreta* (Cockerell, 1896)	*Arundinaria*, *Phyllostachys* spp., *Pseudosasa*, *Sasa* spp. (Suh and Hodges 2007, Kang et al. 2008, Suh 2020)	Sap-sucking
*Unachionaspis tenuis* (Maskell, 1897)	*Arundinaria simonii*, *Bambusa*, *Phyllostachys* spp., *Pleioblastus*, *Sasa*, *Shibataea* spp. (Suh and Hodges 2007, García-Morales et al. 2016, Suh 2020)	Sap-sucking
** Eriococcidae **		
*Acanthococcus onukii* (Kuwana, 1902)	*Bambusa*, *Phyllostachys nigra*, *Pseudosasa*, *Sasa* (García-Morales et al. 2016, Suh 2020)	Sap-sucking
** Miridae **		
*Erimiris tenuicornis* Miyamoto & Hasegawa, 1967	*Sasa* sp. (Kerzhner 1988, Ahn et al. 2018)	Sap-sucking
** Pentatomidae **		
*Aenaria lewisi* (Scott, 1874)	Bamboo (Yasunaga et al. 1993)	Sap-sucking
*Brachymna tenuis* Stål, 1861 (new record)	Bamboos (*Phyllostachys*, *Semiarundinaria*) (Huang and Liu 2006, Zhao et al. 2006, Shu and Wang 2015, Zhang and Zuo 2015)	Sap-sucking (Shu and Wang 2015)
** Pseudococcidae **		
*Antonina crawi* Cockerell, 1900	*Arundinaria* spp., *Bambusa*, *Indocalamus herklotsii*, *Phyllostachys* spp., *Pleioblastus*, *Pseudosasa* spp., *Sasa* spp., *Semiarundinaria fastuosa* (García-Morales et al. 2016, Suh 2020)	Sap-sucking
*Antonina nakaharai* Williams & Miller, 2002	*Arundinaria simonii*, *Phyllostachys* spp. (Lee and Suh 2011, Suh 2020)	Sap-sucking
*Brevennia pulveraria* (Newstead, 1892)	*Sasa* (García-Morales et al. 2016)	Sap-sucking
*Trionymus hamberdi* (Borchsenius, 1949)	Various bamboos including *Pseudosasa japonica* (Li et al. 2014, Ülgentürk et al. 2014, García-Morales et al. 2016, Suh 2020)	Sap-sucking
*Palmicultor lumpurensis* (Takahashi, 1951)	Various bamboos (Li et al. 2014, Ülgentürk et al. 2014, García-Morales et al. 2016)	Sap-sucking
*Pseudococcus comstocki* (Kuwana, 1902)	*Sasa borealis* (García-Morales et al. 2016, Suh 2020)	Sap-sucking
** HYMENOPTERA **		
** Tenthredinidae **		
*Tenthredo nigropicta* (Smith, 1874)	*Pleioblastus* spp. (Shinohara and Ibuki 2018)	Defoliator (Shinohara and Ibuki 2018)
** LEPIDOPTERA **		
** Crambidae **		
*Circobotys aurealis* (Leech, 1889)	*Bambusa* spp., *Phyllostachys* spp., *Pleioblastus* spp. (Haoje et al. 2002)	Leaf-roller (Shu and Wang 2015)
*Crypsiptya coclesalis* (Walker, 1859) (not confirmed)	*Arundinaria, Bambusa* spp., *Dendrocalamus* spp., *Kinabaluchloa wrayi*, *Phyllostachys* spp., *Schizostachyum pergracile*, *Thyrsostachys oliveri* (Sibuea et al. 2020)	Leaf-roller (Sibuea et al. 2020)
*Demobotys pervulgalis* (Hampson, 1913)	Bamboo (Shu and Wang 2015)	Leaf-roller (Shu and Wang 2015)
*Sinibotys butleri* (South, 1901)	*Phyllostachys* spp. (Lee et al. 2019)	Possibly leaf-roller (not confirmed)
*Sinibotys evenoralis* (Walker, 1859)	*Bambusa* spp., *Phyllostachys* spp., *Pleioblastus* spp. (Kim and Lee 1986, Haoje et al. 2002, Robinson et al. 2010)	Leaf-roller (Kim and Lee 1986)
** Erebidae **		
*Amata germana* Felder, 1862	Bamboo (Kishida 2011b)	Probably defoliator
*Rivula aequialis* (Walker, 1863)	Bamboo (Kononenko and Pinratana 2013)	Probably defoliator
*Rivula sericealis* (Scopoli, 1763)	Bamboo (Kishida 2011b)	Probably defoliator
** Lasiocampidae **		
*Euthrix albomaculata* (Bremer, 1861)	Bamboo (Robinson et al. 2010, Kishida 2011a)	Probably defoliator
** Noctuidae **		
*Bambusiphila vulgaris* (Butler, 1886)	Bamboos; *Phyllostachys aurea*, *Pleioblastus hindsii* (Yoshimatsu et al. 2005, Kang et al. 2008)	Bamboo-shoot cutworm (Hill 2008, Kang et al. 2008)
*Triphaenopsis jezoensis* Sugi, 1962	Dwarf bamboo (Keiko et al. 2012)	Florivory, larvae feeding spikelets and caryopses (Keiko et al. 2012)
*Triphaenopsis lucilla* Butler, 1878	Bamboo (Sugi 1987, Choi 2008)	Probably defoliator
** Notodontidae **		
*Mimopydna pallida* (Butler, 1877)	*Sasa* (Robinson et al. 2010)	Probably defoliator
** Zygaenidae **		
*Artona martini* Efetov, 1997	Bamboos (Byun et al. 2010, Marianelli et al. 2020)	Defoliator (Byun et al. 2010)
*Balataea gracilis* (Walker, 1865)	Bamboo (Hirowatari et al. 2013)	Probably defoliator
*Balataea octomaculata* (Bremer, 1861)	Bamboo (Hirowatari et al. 2013)	Probably defoliator
*Fuscartona funeralis* (Butler, 1879)	Bamboos (Kang et al. 2008, Hirowatari et al. 2013)	Defoliator (Kang et al. 2008)
** ORTHOPTERA **		
** Tettigoniidae **		
*Conocephalus bambusanus* Ingrisch, 1990	*Pseudosasa* spp. (Kim and Kim 2002)	Probably defoliator
*Palaeoagraecia lutea* (Matsumura & Shiraki, 1908)	*Pseudosasa* (Kim and Lee 2019)	Probably defoliator
